# Synthetic and Natural Fiber-Reinforced Polymer Matrix Composites for Advanced Applications

**DOI:** 10.3390/ma15176030

**Published:** 2022-09-01

**Authors:** M. R. M. Asyraf, T. Khan, A. Syamsir, A. B. M. Supian

**Affiliations:** 1 Engineering Design Research Group (EDRG), School of Mechanical Engineering, Faculty of Engineering, Universiti Teknologi Malaysia (UTM), Johor Bahru 81310, Johor, Malaysia; 2 Centre for Advanced Composite Materials (CACM), Universiti Teknologi Malaysia (UTM), Johor Bahru 81310, Johor, Malaysia; 3 Department of Engineering Management, College of Engineering, Prince Sultan University, Riyadh 11586, Saudi Arabia; 4 Institute of Energy Infrastructure, Universiti Tenaga Nasional, Jalan IKRAM-UNITEN, Kajang 43000, Selangor, Malaysia

“*Synthetic and Natural Fiber Reinforced Polymer Matrix Composites for Advanced Applications*” is a recently opened Special Issue (SI) of *Materials* that focuses on the fundamentals, characterization, and applications of fiber-reinforced polymer composites. This SI intends to publish reviews and research articles on the new scientific, applied investigations, product development, and lifecycle analysis of enhanced synthetic and natural fiber-reinforced polymer composites for advanced applications.

Fiber-reinforced polymer (FRP) composites are an emerging type of material employed by various sectors to substitute heavy and expensive conventional materials to produce lightweight products. From this point of view, FRP composites have been considered hugely important among researchers because of their versatile properties, applications, and fabrication process. Thus, this composite has been applied in most engineering industries, including defense [1], civil and construction [2], medical and health sciences [3], aerospace [4], and naval [5]. In recent years, many technological achievements have been accomplished by researchers through research activities, including the characterization, design development, computational analysis, and prototype development on FRP composites. These activies would contribute to advancing this exciting material to realize fast-growing global demands. FRP composites have two primary constituents: the fibers act as reinforcement and are embedded in polymer matrices. These fibers can be synthetic materials (aramid, glass, and carbon) or natural materials from minerals, animals, and plants [6,7]. The polymer matrices are categorized into two type: thermoplastics and thermosetting. Thermoplastic materials such as polymers are considered one of the dominant matrices, since they are usually applied for biofibers and can be recycled. These thermoplastic polymers include polypropylene (PP), polyethene, and polyvinyl chloride (PVC). Phenolic, epoxy, and polyester resins are the most commonly used thermosetting matrices, and they are primarily employed in structural materials because they possess high strength properties [8].

In general, synthetic FRP composites have remarkable properties over monolithic polymer materials, such as long creep, fatigue life, and adaptability to the proposed product functions [9,10]. Furthermore, synthetic FRP composites can also be fabricated with fillers to make them multifunctional materials with various properties. Moreover, these synthetic composites can be enhanced with additional improvements regarding corrosion and wear resistance, environmental stability, thermal insulation, and conductivity [11]. Recently, a wide range of structural engineering employed synthetic FRP composites such as a reinforcement material in strengthening walls [12], FRP composite structural systems [13], reinforcing rods and tendons [14], composite bridge decks [15], and wraps for seismic retrofit of columns [16].

Natural fiber-based composites are composed of natural fibers reinforced either in thermoplastic or thermoset composites. Generally, the natural fiber composites offer various ecological values and technical merits, such as low cost, wide availability, and low energy consumption. These values led the global community to change their interest in environmentally friendly materials. These environmentally friendly materials include natural fibers, biopolymers, and biocomposites (natural fiber composites). Biocomposites are mainly manufactured from plant fibers such as flax, hemp, wood, sugarcane, bamboo, grass, kenaf, sisal, coir, sugar palm, banana, and pineapple leaf fibers [17,18]. However, biocomposites have several demerits. These demerits include poor interfacial bonding and the hydrophilic property of lignocellulosic fibers. In addition, the high concentration of hydroxyl groups makes the lignocellulosic fibers hydrophilic, causing them to have poor bonding with matrices [19]. This leads to a lack of mechanical, thermal, and physical performance. Thus, fiber modifications are proposed to solve this issue via chemical or physical treatments [20]. Another practical solution to this problem is employing nanofillers in biocomposites. This effort can improve mechanical, optical, electrical, and magnetic properties.

Even though synthetic FRP composites exhibit excellent mechanical performance, these materials have some issues, namely high raw material cost, poor recycling, high density, and non-biodegradability. From this perspective, biocomposites have emerged as a new biomaterial with good mechanical strength, making them a viable option for replacing synthetic FRP composites. Furthermore, natural fibers currently have various advantages compared to synthetic fibers, making them attractive as reinforcement materials in composite technology. In this case, natural fibers were usually obtained from abundant renewable resources, allowing continuous supply and substantial cost savings for composite sectors.

This Special Issue captures cutting-edge, state-of-the-art research on the recent findings and the technological development of synthetic and natural FRP composites for advanced applications. The topic themes cover recent achievements in terms of modifications of synthetic and natural fibers, characterization techniques of FRP composites, additive manufacturing processes, manufacturing processes, design and product development, material selections, and structure–property relationships.

## Data Availability

All information are available within the articles.
